# Prevalence of *Giardia duodenalis* Infection in Household Cats of Ahvaz District, South-West of Iran

**Published:** 2010-09

**Authors:** B Mosallanejad, R Avizeh, MH Razi Jalali, AR Alborzi

**Affiliations:** 1 Department of Clinical Sciences, Faculty of Veterinary Medicine, Shahid Chamran University of Ahvaz, Ahvaz, Iran; 2 Department of Pathobiology, Faculty of Veterinary Medicine, Shahid Chamran University of Ahvaz, Ahvaz, Iran

**Keywords:** Giardia *duodenalis*, Prevalence, Cat, Iran

## Abstract

**Background:**

The occurrence of *Giardia duodenalis* in cats is of potential significance from both clinical and public health perspectives. The object of this study was antigenic detection of *G. duodenalis* in household cats of Ahvaz district, South-West of Iran.

**Methods:**

The prevalence of *G. duodenalis* was determined in fecal samples by two techniques: centrifugation-flotation and a commercial *Giardia* Antigen Test Kit (immunochromatography assay) in 150 household cats of different ages among referred cases to Veterinary Hospital of Ahvaz University from January 2008 to February 2010.

**Results:**

Five out of 150 fecal samples (3.33%) were positive for antigen of *G. duodenalis* by immunochromatography assay. The prevalence was significantly higher in young cats less than 6 months (15.79%) compared with adult cats 6 months – 3 years (1.37%) (*P*=0.027) and above 3 years (1.72%) (*P*=0.044). The infection had more prevalence in diarrheic cats (17.39%) compared with non-diarrheic cats (0.79%) and the difference was significant (*P*=0.02) as well. The prevalence was higher in male cats (3.41%) than females (3.23%) and in the season of autumn (6.06%), but the difference was not significant between the prevalence of infection relative to host gender and season (*P*>0.05). Microscopy examination on fecal samples showed that 2% of the studied cats were positive.

**Conclusion:**

The parasite antigen was present as a zoonotic infection in Ahvaz district, South-west of Iran. More sensitive techniques, such as immunochromatography assay, might yield more reliable results, in the detection of low levels of *Giardia* in fecal samples of cats.

## Introduction

*Giardia duodenalis* is a protozoan parasite, which is found in the small intestine of vertebrates including mammals (1). Only the species *G. duodenalis* (also known as *G. intestinalis* and *G. lamblia*) has been recognized in cats (2). Most infections are subclinical or show only transient softening of the stool early in the infection, although diarrhea may be acute, chronic, or intermittent in dogs and cats. Clinical signs are most likely to be seen in younger animals from multi-cat households (3–6). Transmission is fecal-oral route by ingestion of feces or fecal-contaminated water, food or fomites. There are two stages in the life cycle. Trophozoites are the active motile form. The trophozoites move towards the colon where they produce a cyst form. The cysts are extremely hard and can survive for long periods in the water. The parasite has a one to two week incubation period. Data on *Giardia* species infection in cats show a similarly wide range of prevalence (0%– 52%) (7–10).

For reliable diagnosis of intestinal parasites, a combination of several techniques has been recommended. The diagnosis of *Giardia* infection traditionally is depended on microscopic identification of trophozoites or cysts in feces of affected animals. Although flotation is a reference method for the detection of *Giardia* cysts, it is suggested that an alternative test is also needed because microscopic examination is time consuming and needs an experienced microscopist. Many artifacts mimic to varying degrees the morphology of *Giardia* cysts (5,11).

Several laboratory methods have been developed to detect antigen in the feces of infected cats such as ELISA, immunofluorescence assay and molecular techniques. Though these tests are more sensitive, specific and more reproducible, but they can be expensive and generally take time to be analyzed by a specialized laboratory. Recently, a commercial *Giardia* Antigen Test Kit (immunochromatography assay) (BVT Co., Ltd, Lion) was released for detection of *G. duodenalis* antigen in feline feces. This test is a rapid enzyme immunoassay that can be conducted on fresh feces or previously frozen feces. Sensitivity and specificity for kits of *Giardia* Ag Test were 95.6% and 100% respectively (12).

Large numbers of cats are found roaming residential streets and increase the risk of public health for other animals and humans. Lack of consideration to the health of these animals can decrease the health of humans. No study has been reported on the distribution of giardiasis in the household cat population in Iran. We conducted this study in order to determine the prevalence of *G. duodenalis* in fecal samples of the household cats in Ahvaz area, southwestern Iran. The results of this study can be important for pet clinicians.

## Materials and Methods

### Study area and sample population

A total of 150 household cats of different ages were examined for fecal antigens of *G. duodenalis* by immunochromatography assay and for cyst or trophozoite in feces by microscopic examination (flotation method). The cats used in this study were referred cases to Veterinary Hospital of Ahvaz University from January 2008 to February 2010. Most of the cats had been referred for other reasons mostly for vaccination. Samples were stored in an ice chest and transported to the Parasitology Laboratory of Veterinary Faculty to be processed.

Three stool samples were collected from each animal at 48-hour intervals, producing a total of 450 stool samples. Information about household cats was taken from their owners. The studied cats were divided into two groups (diarrheic and non-diarrheic) and based on age into three groups (group 1: <6 months, group 2: 6 months – 3 years and group 3: >3 years). Classification was made by sex, breed, and season. Age was estimated by dental formulary and owner information.

### Laboratory methods

Two methods of fecal centrifugation-flotation technique and immunochromat-ography assay (IC) were employed.

### Fecal centrifugation-flotation technique

Fecal samples (1 g) of 150 cats (×3) were examined microscopically for the presence of *G. duodenalis* cysts by flotation in 33% zinc sulphate solution (specific density 1.27). It was filtered through gauze, and centrifuged in a 15 ml tube at 400 g for 10 min. A drop of the float from the meniscus was examined microscopically at 400x magnification for the presence of *G. duodenalis* cyst (13).

Trophozoites could not be detected by floatation techniques because the floatation solution lyses the trophozoites, so direct fecal smears were carried out for demonstration of trophozoites. Three samples were collected at 48-hours intervals, because of the intermittent nature of *Giardia* shedding.

### Immunochromatography assay and interpretation of the test

**Fig. 1:**
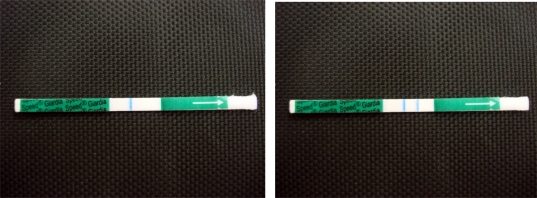
Positive (Left) and negative (Right) samples of rapid *Giardia* Ag test in household cats in Ahvaz district, Iran by ICA, 2008–2010

Fecal samples were collected from the studied cats using the sample collection. We added a volume of 1 full spoon of fecal sample into the buffer diluent. Then the vial was closed and shaken for homogenization. We placed label on the sample tube for identification and took out the sample spoon, provided with the kit. The strip was let for one minute in the solution. Then it was removed and placed on a flat and horizontal surface for migration. Rapid detection of soluble *G. duodenalis* cyst antigens (BVT Co., Ltd, Lion) is a qualitative test. A positive result indicates the presence of *Giardia* cysts in the feces. One blue and one colored line are positive (Fig. 1). One blue colored line was negative (Fig. 1). Speed *Giardia* helps us detecting the cyst presence for a concentration higher than 80 cysts per gram of feces (12).

### Statistical analysis

Cats were grouped by age, sex, breed, and season and diarrheic or non-diarrheic to determine whether these factors were associated with *G. duodenalis* infection, using Chi-square analysis, Fisher's exact test, and Z test. Statistical comparisons were carried out using SPSS 16.0 statistical software. Differences were considered significant when *P*<0.05.

## Results

Five out of 150 fecal samples (3.33%) were positive for antigen of *G. duodenalis* by immunochromatography assay. The prevalence was significantly higher in young cats less than 6 months (15.79%) compared with adult cats 6 months–3 years (1.37%) (*P*=0.027) and above 3 years (1.72%) (*P*=0.044). The infection had more prevalence in diarrheic cats (17.39%; 4 out of 23) compared with non-diarrheic cats (0.79%; 1 out of 127) and the difference was significant (*P*=0.02). The prevalence was higher in male cats (3.41%) than females (3.23%) and in the season of autumn (6.06%), but the difference was not significant between the prevalence of infection relative to host gender and season (*P*>0.05). Microscopy examination on fecal samples showed that 2% (3 out of 150) of the studied cats were positive. Prevalence in other seasons (winter, spring, and summer) was 4.26%, 2.44%, and 0% respectively. Most of the studied cats were Domestic Short Hair (DSH). All of the affected cats had access to open environment.

## Discussion

The results highlight the potential role of household cats for zoonotic transmission of giardiasis. The present survey that is the first report on the prevalence of *G. duodenalis* in household cats in Iran revealed that the overall prevalence of the infection was 3.33% and 2% by using immunochromatography assay and fecal centrifugation-flotation technique respectively.

The current study demonstrated that *G. duodenalis* infection was significantly more common in cats under 6 months of age compared with adult cats (*P*<0.05). Data examining age as a risk factor for individual pathogens in cats are limited (10,14). The Immaturity is considered a significant risk factor for giardiasis in humans and dogs, suggesting that immunity develops with age (15). Similarly, cats may develop immunity to giardiasis and have no or decreased cyst production after cyst challenge (5). Development of humoral immunity with age may have contributed to the lower prevalence seen in mature cats in this study. Kitten behavior, particularly the habit of biting and licking objects, which can be contaminated with *Giardia* cysts, may also be a significant contributing factor.

Diarrhea has been reported as the major clinical disease for *Giardia* infection (16,17). However, subclinical infection is likely to be also common (18). Interestingly, diarrhea was an important sign of infection in the present study, because the prevalence of infection was 17.39% in diarrheic cats, compared with non-diarrheic cats (0.79%) and the difference was significant (*P*=0.02).

Generally, a centrifugation–flotation technique is regarded as more sensitive and accurate than flotation only to detect protozoan cysts (19). For the detection of *Giardia*, immunochromatography assay is more sensitive than centrifugation–flotation (12). For these reasons, feces of cats were examined by centrifugation–flotation technique and immunochromatography assay in the present survey. It is shown that the estimated amount of *Giardia* antigens can widely range in positive stool samples, for instance above 80 cyst in 1 gram faeces in samples found positive to *Giardia* by immunochromatography assay. Rapid *Giardia* test kit appears to be sufficiently sensitive to detect cases of giardiasis when fairly high levels of antigen are shed.

Surveys in many countries have shown that infection with *Giardia* is common and widespread in crowded and open environments. Higher prevalence in dense populations may be expected because of increased ease of transmission (20,21). Similarly, a high prevalence of *Giardia* species (50%) was detected in a Persian cattery (8). In our study, all of the affected cats had access to open environment.

The higher prevalence was seen in male household cats than females in the present study. These results can be explained by the territorial habits, as males have a wider area of operation than females, of course the difference was not significant between different sexes (*P*>0.05). Generally, sex does not seem to be a determining factor of infection. Regarding seasonal variation in the prevalence of *Giardia*, seasonal effects on the infection rate may reflect climatic changes on the parasite, host physiology or in the photoperiod (15,20). In the present study, the difference was no significant for season changes (*P*>0.05).

The prevalence of *G. duodenalis* infection detected in cats in the present study is slightly higher compared with previous studies in stray cat population in Iran. Arbabi (22) reported 0.9% (1 out of 113 samples) infection in stray cats in Kashan.

The prevalence of giardiasis has been studied on dogs in some different areas of Iran. Razmi (23) reported 1.1% infection with study on 174 fecal samples, Jafari Shoorijeh et al. (24) (0.68%) and Shirani et al. (25) (3.33%). There is no obvious explanation for these differences. These may be due to geographical variation or to differences in the number of animals and type of population surveyed, or may be attributed to different sensitivity of the diagnostic procedure used.

There are many studies of the general prevalence of giardiasis in cat populations worldwide. Data on *Giardia* species infection in cats show a similarly wide range of prevalence (0– 52%) (7–10). Studies conducted had recorded prevalence of 2.4%- 7.3%, in cats in the Perth Metropolitan area (16), 4% in Unites State (26), 0.9% in Japan (27), 14% in Western Australia (2) and 16% in Sydney (28).

Excretion of *G. duodenalis* cysts is intermittent in symptomatic and asymptomatic animals (21), so one negative fecal exam may not necessarily mean that the animal is not parasitized by these protozoans. Diagnosis can be improved repeating examinations whenever possible. Mundim (28) found that examination of three samples from the same animal increased the likelihood of positive results. In the present study, three samples were collected from each cat at 48-hour intervals, due to intermittent excretion of trophozoites and cysts. The methods used for detection of parasites are likely to play a great role in the variable prevalence detected worldwide. More levels of immunochromatography positives and lack of confirmation by microscopy may be due to low numbers of cysts in fecal samples and microscopy was not sensitive enough to detect these low levels. Similar observations have been reported by Collins (29). Trophozoites rapidly disintegrate in feces over time, thus reducing the likelihood of microscopic identification (15). In our survey, two samples were positive on the immunochromatography test, but negative by microscopy examination.

In conclusion, this is the largest study of its kind investigating prevalence of *G. duodenalis* infection in household cats with alimentary signs. Due to close contact of cats with human and this fact that children play outdoors on the soil, cats can be an important potential source of transmission of zoonotic parasite such as *Giardia*. They have an important role in contamination of environment to cyst or trophozoite. Our results indicated that the parasite antigen was present in Ahvaz district, South-west of Iran. It is suggested that climatic conditions in this area (warm and humid) are relatively suitable for the spread and survival of the cysts. It is possible that household cats are a potential source of environmental contamination in Ahvaz area. Prevalence data are an essential component for evaluation of zoonotic risk.

Our results will be the basis of further studies that will permit to deepen our knowledge of the epidemiology of giardiasis. Further studies in various areas will be necessary to survey the overall epidemiological status of giardiasis in household and stray cat populations.
